# Mid- to Long-Term Outcomes of Two-Stage Revision Arthroplasty for Periprosthetic Joint Infection of the Shoulder

**DOI:** 10.3390/jcm14020547

**Published:** 2025-01-16

**Authors:** Ağahan Hayta, Doruk Akgün, Anh Do, Rony-Orijit Dey Hazra, David Alexander Back, Nihat Demirhan Demirkiran, Markus Scheibel, Alp Paksoy

**Affiliations:** 1 Center for Musculoskeletal Surgery, Charité Universitätsmedizin Berlin, 13353 Berlin, Germany; 2 Department of Orthopedics and Traumatology, Kütahya Health Sciences University, 43020 Kütahya, Türkiye; 3 Department for Shoulder and Elbow Surgery, Schulthess Clinic, 8008 Zurich, Switzerland

**Keywords:** shoulder arthroplasty, periprosthetic joint infection, RTSA

## Abstract

**Background/Objectives**: Periprosthetic joint infection (PJI) after shoulder arthroplasty is often treated with a two-stage approach, but the data on the mid- to long-term outcomes remain scarce. This study aimed to evaluate the clinical outcomes of two-stage revision arthroplasty for shoulder PJI with a minimum follow-up of five years. **Methods**: This retrospective study identified 59 shoulders in 58 patients who underwent the first stage of a two-stage revision arthroplasty for shoulder PJI at our institution between 2007 and 2018. Of these, 29 shoulders in 29 patients (49.2%) did not undergo reimplantation or the patient passed away before reaching five years of follow-up. The remaining 30 shoulders in 29 patients were included in the study. The clinical assessments included the active range of motion, the visual analogue scale (VAS) for pain, the Subjective Shoulder Value (SSV), the Constant Score (CS), and the 12-Item Short Form Survey (SF-12), supplemented by detailed clinical and radiological evaluations. **Results**: The mean age of the 29 patients was 75.9 ± 10.4 years. The average follow-up duration was 8.3 ± 2.8 years. The most common indications for primary shoulder arthroplasty were primary osteoarthritis (n = 12, 40%) and fractures (n = 12, 40%). At the first stage, nine cases (30%) showed negative cultures, while *C. acnes* and *S. epidermidis* were each identified in eight cases (26.7%). Four shoulders (13.3%) experienced recurrent infections. At the follow-up, the mean abduction was 86 ± 48.1°, the mean forward flexion was 97.8 ± 50.1°, the mean external rotation was 20.5 ± 19.9°, and the internal rotation reached the lumbosacral region. The mean VAS pain score was 1.5 ± 2.1, the mean SSV was 51.8 ± 28.4%, the mean CS was 54.6 ± 21.0, and the mean SF-12 was 81.0 ± 16.0. **Conclusions**: Two-stage revision arthroplasty for shoulder PJI results in satisfactory subjective and objective outcomes, with a low overall reinfection rate. However, the high rates of mortality and failure to reimplant must be carefully considered when managing expectations in this challenging cohort.

## 1. Introduction

Periprosthetic joint infection (PJI) of the shoulder is one of the most challenging complications, estimated to occur in around 1–2% of cases after primary arthroplasty and up to approximately 15% of cases after revision arthroplasty [1,2,3]. With a rising number of shoulder arthroplasties being performed, and as a result of the growing elderly population, a subsequent increase in PJI has to be expected, which underscores the need to treat PJI accurately to achieve lasting infection-free survival [4,5].

The most commonly used treatment options include long-term antibiotic suppression [6,7], debridement with antibiotics and implant retention [8,9], resection arthroplasty [8], arthrodesis [10], and one- or two-stage revision [11]. Although two-stage revision is considered the treatment of choice for shoulder PJI, there is no consensus regarding the relative indications for single- or two-stage revision. Recent meta-analyses indicate that one-stage revision can be as effective as two-stage revision in a selected group of patients to reduce the need for additional surgery, the costs, and the risks for patients [11,12,13]. A considerable subset of patients never undergo the second stage for various reasons, including mortality, medical comorbidities, or the patient’s unwillingness to undergo a second surgery [14]. However, patients with highly virulent and multidrug-resistant microorganisms or chronic infections tend to be admitted more often to the two-stage operation, as it is not limited by the previous identification of the causative organism or soft-tissue conditions and is associated with potentially better infection control [15]. Single- and two-stage revisions have been reported to result in the eradication of infection in more than 90% of cases of PJI at short-term follow-up [16,17,18,19], with the longest reported mean follow-up being five years [20,21,22,23]. To our knowledge, there are only three studies that have evaluated the outcomes of two-stage revision for PJI wherein patients had a minimum follow-up of five years [20,22,24].

Given this lack of data on the long-term outcomes of this approach, there is a need for further evidence regarding the efficacy of two-stage revision. Therefore, the aim of the present study is to contribute to the published literature pertaining to shoulder PJI management by presenting our experience of two-stage revision arthroplasty with respect to the presenting features of the infected joint, the microbiological profile, the eradication of infection, and the reoperation rates.

## 2. Materials and Methods

### 2.1. Study Design and Cohort

This study received approval from our institutional ethics committee (Approval number EA4/040/14). A total of 59 shoulders in 58 patients (31 females, 27 males; mean age: 78.5 ± 11.3 years) who were admitted to two-stage revision for shoulder PJI between 2007 and 2018 were identified. Each patient identified was indicated for revision with suspicion for infection or positive preoperative or intraoperative cultures. The inclusion criteria were a completed two-stage revision arthroplasty with reimplantation and a follow-up duration of at least five years. Of the initial 58 patients, in 29 (50%) there was failure to reimplant or the patient passed away before reaching the minimum five-year follow-up duration. Thus, 30 shoulders in 29 patients were included. Of these, 20 shoulders in 19 patients were available for clinical follow-up. Ten patients could not be brought in for the clinical follow-up; therefore, their follow-up data from routine examinations were retrospectively reviewed. The follow-up data for the deceased patients and the patients who did not undergo reimplantation are presented separately in the Results Section.

### 2.2. Data Collection and PJI Definition

The included patients’ electronic medical records were reviewed, and the patients’ demographics, comorbidities, preoperative diagnoses, previous treatments, surgical details, subsequent revisions, time to revision, laboratory values including serum C-reactive protein (CRP) and blood leukocyte count, microbiology data, and pathology data were extracted.

The patients were retrospectively classified based on their medical records at the time of the initial presentation with a suspected infection, using criteria from the latest proposed definition for shoulder PJI by the International Consensus Meeting (ICM) [25]. Each patient was assessed using the specific criteria outlined by the ICM, which included factors such as the presence of a sinus tract, intra-articular purulence, the identification of virulent or non-virulent organisms, wound drainage, humeral loosening, positive frozen section results, preoperative aspiration with polymorphonuclear neutrophil percentages, elevated erythrocyte sedimentation rate (ESR), increased CRP, and cloudy synovial fluid. The patients were then classified into one of the following categories: (1) definite PJI, (2) probable PJI, (3) possible PJI, or (4) PJI unlikely. Additionally, the data on Elixhauser comorbidities at the time of admission for surgery were retrieved from the electronic medical records and coded using the International Classification of Diseases (ICD-10) system [26].

The criteria for successfully treating shoulder PJI in terms of infection eradication were derived from the Delphi-based international multidisciplinary consensus as described in various publications [14,27,28,29]. The eradication was deemed successful if, at the latest follow-up, the following criteria were met: (a) infection eradication, indicated by a healed wound without pain, fistula, or drainage and no recurrent infection; (b) no PJI-related mortality; (c) no subsequent surgical intervention for infection post-reimplantation surgery; and (d) no long-term (over six months) antimicrobial suppression therapy.

### 2.3. Two-Stage Revision Arthroplasty Protocol

All revision surgeries were performed exclusively at our institution following a standardized two-stage revision arthroplasty protocol. The first stage involved the removal of all prosthetic components, sutures, cement, and infected tissue. The scar tissue was carefully dissected, with the precise release and mobilization of the remaining rotator cuff. This was followed by a thorough debridement and irrigation using high-pressure pulsatile lavage. A custom-made 0.5 g gentamicin-loaded antibiotic cement spacer was then implanted. In all cases, the deltopectoral approach was consistently utilized.

For each patient, various samples were obtained from the synovial fluid, infection-suspicious tissue, the pus (if applicable), and the periprosthetic membranes for microbiological and histological examinations. The cultures were maintained for two weeks to detect slow-growing pathogens, such as *Cutibacterium* species. The histopathologic analysis of the intraoperatively taken samples was conducted based on the consensus classification of the periprosthetic membrane and neo-synovium (formerly called the “synovial-like interface membrane”) [30,31,32]. The prosthesis components were sent for sonication to improve the microbiological diagnosis [33]. The sonication was carried out for one minute at 40 kHz using a BactoSonic 14.2 unit (Bandelin, Berlin, Germany), following the established protocol [34]. The sonication fluid obtained was subsequently cultured on both aerobic and anaerobic sheep blood agar plates and then incubated for a period of 14 days.

A tailored antibiotic regimen, recommended by our infectious diseases department based on previous publications, was administered postoperatively to all patients [14,35,36,37]. This consisted of two weeks of intravenous antibiotic therapy and subsequent oral antibiotic therapy until reimplantation. In the event of persistent infection, indicated by a discharging wound, local signs of infection, and/or increasing CRP without any other focus, an additional revision involving irrigation, debridement, and concurrent spacer exchange (“three-stage exchange”) was executed between the two stages.

The second-stage reimplantation was performed once the surgical site had healed and was deemed suitable based on the condition of the soft tissues, without adhering to a fixed minimum interval after the first stage. The procedure was conducted using the same deltopectoral approach. This stage also provided an opportunity for the additional thorough debridement of the surrounding soft tissues and bone prior to the reimplantation of the definitive components. The choice of implant was determined by the quality of the remaining bone stock and the condition of the rotator cuff tendons. Following the reimplantation, the patients received individualized intravenous antibiotic therapy for one to two weeks; they were then transitioned to oral antibiotics to complete a total treatment duration of at least six weeks after the reimplantation.

### 2.4. Clinical Follow-Up

The active range of motion (ROM) was measured with a goniometer and documented. The visual analogue pain scale (VAS) was used to assess the pain levels. As part of the patient-reported outcome measures, the Constant Score (CS) and the Subjective Shoulder Value (SSV) were employed to evaluate the postoperative shoulder-related outcomes, while the 12-Item Short Form Survey (SF-12) was utilized to assess the impact on an individual’s everyday life. Standard anteroposterior and lateral scapula view radiographs were obtained preoperatively, postoperatively, and at the last follow-up to evaluate loosening, osteolysis, or changes in the implant’s position. The radiological evaluation was carried out by three authors: A.H., A.P., and D.A.

### 2.5. Statistical Analysis

The IBM SPSS Statistics software (version 29.0; IBM, Armonk, NY, USA) was utilized for the statistical analyses. Descriptive statistics were performed, and the results were presented as the mean and standard deviation or as numbers and percentages. The chi-square test was used to measure the differences between the categorical variables. The differences between the continuous variables were measured with the *t*-test or one-way ANOVA. *p*-values < 0.05 indicated statistical significance unless otherwise stated.

## 3. Results

### 3.1. First Stage

In the examined group (30 shoulders in 29 patients), the mean age of the patients was 75.9 ± 10.4 years, with more females (n = 18, 62.1%) than males. The mean follow-up was 8.3 ± 2.8 years after the reimplantation. The indications for primary shoulder arthroplasty included primary osteoarthritis (n = 12, 40%), fractures (n = 12, 40%), post-traumatic osteoarthritis (n = 2, 6.7%), infection-related osteoarthritis (n = 2, 6.7%), instability-related osteoarthritis (n = 1, 3.3%), and post-traumatic necrosis of the humeral head (n = 1, 3.3%).

After applying the ICM criteria for the diagnosis of shoulder PJI, nine cases were classified as “PJI unlikely”, three as “possible PJI”, thirteen as “probable PJI”, and five as “definite PJI”. A comparison of the baseline parameters such as age, gender, BMI, the timing of infection, and Elixhauser comorbidities revealed no statistically significant differences between these diagnostic groups. However, significant differences were observed when comparing types of prior hardware, particularly with reverse total shoulder arthroplasty (RTSA) (*p* < 0.001) and cases where a spacer had already been implanted at the first presentation (*p* = 0.05).

The time from the initial surgery to the revision was categorized based on the classification by Sperling et al. as acute (<3 months), sub-acute (3–12 months), or chronic (>12 months) [2]. The majority of the cases in this cohort were chronic (22 cases, 73.3%), followed by sub-acute (7 cases, 23.3%) and acute (1 case, 3.3%). Table 1 summarizes the four diagnostic groups classified according to the ICM criteria, along with the onset of infection, the Elixhauser comorbidities, and the baseline demographics.

Concerning the type of existing arthroplasty at the time of explantation, fourteen shoulders had hemiarthroplasty (HA), seven shoulders had anatomical total shoulder arthroplasty (ATSA), and six had RTSA. Three shoulders had no prosthesis at their first presentation, as the infected prosthesis had already been explanted at an outside institution.

The mean time from the primary arthroplasty to explantation was 4 ± 4 years (range, 0.1–14). The mean preoperative serum CRP levels at the first stage were 11.3 ± 14.6 mg/L. For each patient, a minimum of three tissue samples (mean: 4.5 ± 1.7) were collected at the first stage for microbiological cultures. Among these samples, *Cutibacterium acnes* and *Staphylococcus epidermidis* were each identified in eight cultures (26.7%). *Staphylococcus capitis* and *Staphylococcus aureus* each grew in two cultures (6.7%). Additionally, *Enterobacter cloacae*, *Enterococcus faecalis*, *Anaerococcus prevotii*, *Pseudomonas aeruginosa*, *Paenibacillus pabuli*, *Staphylococcus hominis*, and *Streptococcus parasanguinis* were each identified in one culture (3.3%). Notably, six cases demonstrated polymicrobial growth (20%), and nine shoulders (30%) were culture-negative. Table 2 provides an overview of the bacteria identified in the cultures.

### 3.2. Second Stage

Between the two stages, three shoulders (10%) underwent arthroscopic biopsy. Additionally, five shoulders (16.7%) required further surgical intervention involving spacer exchange. Specifically, two shoulders (6.7%) underwent one additional spacer exchange, and one shoulder (3.3%) underwent two additional spacer exchanges due to persistent infection. Moreover, two shoulders (6.7%) required bony augmentation due to poor glenoid bone stock.

**Figure 1:**
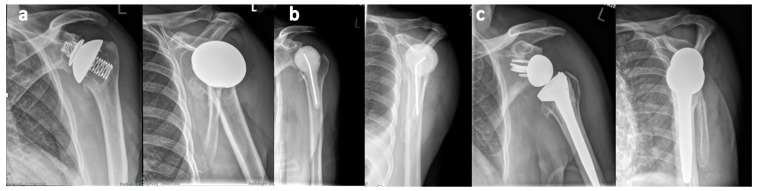
(**a**) Suspicion of low-grade infection with humeral component loosening after the implantation of an ATSA in a 70-year-old patient from our cohort with persistent pain and stiffness. The initial diagnostic arthroscopy did not reveal any culture evidence of infection. (**b**) At the first stage, the ATSA was explanted, the suspicious tissue suggestive of infection was removed, and an antibiotic spacer was implanted. The intraoperatively obtained tissue samples grew *Cutibacterium acnes*. (**c**) Following targeted antibiotic therapy, the implantation of an RTSA was performed at the second stage.

The reimplantation was performed in 30 shoulders after a mean duration of 10.8 ± 7.4 weeks (range, 1.7–34 weeks) following explantation. The microbiological cultures taken intraoperatively at the time of reimplantation were all negative. The implant designs chosen for the second stage included RTSA in twenty-six shoulders, ATSA in three shoulders, and HA in one shoulder. Figure 1 displays radiographs obtained during the course of two stage revision.

### 3.3. Complications

Four shoulders (13.3%) experienced recurrent infection, with two cases diagnosed within three months after reimplantation and two cases diagnosed approximately ten years after reimplantation. It is noteworthy that the remaining 26 cases (86.7%) demonstrated no requirement for further revision surgery. Additionally, one patient (3.3%) sustained a traumatic periprosthetic fracture after the two-stage revision, which was treated conservatively at an outside institution.

### 3.4. Clinical Follow-Up

Among the 20 shoulders available for the clinical follow-up, the mean abduction was 86 ± 48.1° (range, 0–165°), the mean forward flexion was 97.8 ± 50.1° (range, 0–165°), the mean external rotation was 20.5 ± 19.9° (range, 0–60°), and the internal rotation reached the lumbosacral transition (range, lateral thigh to the 12th thoracic vertebra). Moreover, the mean pain level on the VAS was 1.5 ± 2.1 (range, 0–7). Regarding the patient-related outcome measures, the mean SSV was 51.8 ± 28.4% (range, 10–95), the mean CS was 54.6 ± 23.9 (range, 11–91.7), and the mean SF-12 was 81.1 ± 16 (range, 51.7–108.7), with a mean physical component summary of 34.4 ± 9.4 (range, 17.2–51.7) and a mean mental component summary of 47.7 ± 9.4 (range, 19.4–65). At the follow-up, one patient had a healed periprosthetic fracture without any signs of infection, and one patient had no implant as the revision prosthesis was explanted due to persistent infection. The remaining 18 shoulders (90%) showed no signs of humeral or glenoid loosening. Table 3 displays an overview of clinical follow-up.

### 3.5. Clinical Course of Deceased Patients

A total of 20 out of 58 patients (34.8%) passed away before reaching the minimum five-year follow-up. The last follow-up for these patients occurred at a mean of 20.2 ± 15.1 months (range: 1–47 months) after the first stage.

Six patients (30%) from this group did not proceed to the second stage due to death within three months following the first stage. Among the 14 patients who underwent reimplantation, two (14.3%) were diagnosed with reinfection. In one case, reinfection was identified two months after the two-stage revision. The implanted RTSA was removed and an antibiotic spacer was implanted. The patient remained infection-free for one year before passing away. In the second case, reinfection was diagnosed three days after the two-stage revision. The patient underwent two septic revisions with component changes and remained infection-free for three years before passing away.

The remaining 12 patients in this group were infection-free at their last follow-up. One patient underwent an open reduction and internal fixation procedure following a traumatic periprosthetic fracture. No other revisions were performed in this group.

### 3.6. Patients Without Reimplantation

In total, 15 out of 58 patients (25.9%) did not proceed to the second stage of the two-stage revision. Six of these patients died within three months following the first stage, preventing reimplantation. For the remaining nine patients, the decision not to proceed with the reimplantation was made collaboratively, taking into account the patients’ willingness, expectations, comorbidities, and soft-tissue conditions. These nine patients were followed for a mean duration of 57.3 ± 27 months (range: 33–103 months). Among them, three experienced reinfection (33.3%) and required additional surgery with spacer exchanges to achieve infection control.

### 3.7. Infection Eradication and Mortality

In the main cohort of 30 shoulders in 29 patients who underwent successful reimplantation and had a minimum follow-up of five years, infection eradication was achieved in 26 shoulders (86.7%), meeting the previously mentioned Delphi criteria for success. However, 20 patients passed away within five years of follow-up, resulting in a five-year mortality rate of 34.5%. The infection-related mortality rate remains unclear, as the cause of death for the deceased patients in our cohort is unknown. Overall, 29 out of 59 shoulders in 58 patients (49.2%) did not proceed to reimplantation or the patient passed away before completing the five-year follow-up period.

## 4. Discussion

Our results demonstrate that patients achieve satisfactory subjective and objective clinical outcomes in the mid to long-term following a successful two-stage revision arthroplasty for shoulder PJI. However, it is important to note the high mortality and failure-to-reimplant rates, which together account for almost half of the cohort. The reinfection rate after reimplantation was 13.3%, which appears relatively low, while the mortality rate of approximately 35% aligns with the current literature. Among the follow-up group, with a mean follow-up of approximately eight years, the mean VAS score was nearly 2, the mean SSV was 52%, and the CS averaged 55 points. Furthermore, the mean SF-12 score of 81 points, combined with a tolerable ROM, indicates favorable outcomes in this elderly population.

Our overall reinfection rate (13.3%) after reimplantation is lower compared to many prior studies, which mostly report at short- to mid-term follow-up. Klingebiel et al. included a cohort of 16 patients (9 of whom were reimplanted to a RTSA following two-stage revision) from 2010 to 2019 with a mean final follow-up of 33.2 months (12–85 months), reporting a 19% reinfection rate [38]. Meshram et al. identified 17 cases between 2005 and 2014 with a minimum follow-up of five years (5–9 years), demonstrating a similar infection rate of 18% at the mid-term follow-up [24]. Aïm et al. conducted a meta-analysis with a minimum follow-up of two years and demonstrated a reinfection rate of 21% after two-stage revisions [11]. Buchalter et al. examined 19 patients between 2000 and 2014 with a mean follow-up of 63 ± 36 months (25–184 months) and also found a relatively high reinfection rate (26%) following two-stage revision [20]. However, another meta-analysis by Garrigues et al. with a minimum two-year follow-up found a comparatively lower reinfection rate following two-stage revision (11.4%) [25]. Similarly, in a cohort of 38 patients with a minimum follow-up of 24 months (mean, 52 ± 34 months), 24 of whom underwent a full two-stage revision, Grubhofer et al. demonstrated a high infection eradication rate of 95% [16]. One of the reasons for the discrepancy between these complication rates could be the complexity of patients with shoulder PJI and the challenges of operating on shoulders that have undergone several surgical procedures. Another reason could be the variety of risk factors for developing complications, like male gender, obesity, American Society of Anesthesiologists (ASA) type 3, smoking, and history of surgery before the arthroplasty [39,40,41], which was not taken into account completely in most of the studies.

The present study illustrated good and satisfactory clinical outcomes after two-stage revision, in which RTSA was used as the definitive arthroplasty at the second stage in most of the cases (26 out of 30, 86.7%). Currently, three studies have reported on the clinical outcomes after two-stage revision surgery for shoulder PJI at a mean follow-up of more than five years [20,22,24]. Ortmaier et al. reported the results of 20 patients between 1998 and 2010 at a follow-up of 24 to 115 months [22]. The mean overall postoperative CS was 42.6 (15–72) points, the mean simple shoulder test (SST) was 5.5 (3–10), the mean VAS was 1.5 (0–4), the average abduction was 62°, and the average forward flexion was 77° (the ranges of abduction and forward flexion were not depicted in the study) [22]. Of the 20 patients, the second-stage revision was the implantation of an RTSA in 12 patients (60%), irrigation and debridement with exchange of the polyethylene inlay in 7 patients (35%), and resection arthroplasty in 1 patient (5%) [22]. In the study by Buchalter et al., the average postoperative American Shoulder and Elbow Surgeons Shoulder Assessment (ASES) score was 69 (32–98), the average postoperative forward flexion was 119° (30–160°), the mean external rotation was 19° (−20 to 40°), and the mean internal rotation was lateral ilium to T12 postoperatively [20]. The implant used at the second-stage surgery was RTSA in 10 (53%) patients, ATSA in 5 (26%) patients, and HA in 4 (21%) patients [20]. Recently, Meshram et al. reported on the mid-term clinical outcomes of 17 cases, in which RTSA was used as the definitive arthroplasty at the second stage for all patients [24]. At the final follow-up, the patients showed an average VAS for pain of 2.7 ± 2.2, an ASES score of 70.3 ± 22.9, an SST score of 6.6 ± 3.9, abduction of 121.8 ± 20.88°, and forward flexion of 116.8 ± 22.58°. This suggests that RTSA as the definitive arthroplasty at the second stage of a two-stage revision for shoulder PJI illustrates good clinical function that can be expected beyond the mid-term follow-up and throughout a longer period of time.

From the initial cohort, half of the patients were excluded because they either did not complete the second stage or passed away before reaching the minimum five-year follow-up period after the two-stage revision. This exclusion introduces a significant bias when reporting the success of this surgical procedure. Moreover, focusing solely on infection eradication overlooks a critical subset of patients who are too frail to undergo further surgery, succumb prematurely, or decline additional procedures due to low functional demands following the implant removal. Shoulder PJI and its treatment carry a high mortality risk, as evidenced by this study’s mortality rate of approximately 35%. Notably, the two-stage revision was successful in only around 44% of the cases at a minimum five-year follow-up, with treatment success defined as completing both stages without death or reinfection. However, the infection-related mortality rate remains unclear, as determining the cause of death was particularly challenging for the patients who passed away outside of the hospital, where the precise details often remained unavailable. This lack of information potentially leads to an overestimation of the treatment’s success rate. These factors underscore the potential bias introduced by excluding these patients and highlight the importance of interpreting the seemingly satisfactory results within this broader clinical context.

Unsurprisingly, one-third of the cases turned out to be culture-negative, and the majority of the culture-positive infections at the first stage were *Cutibacterium acnes*. In the prior literature, this Gram-positive, anaerobic bacillus with low virulence characteristics is reported as the most common bacterium in shoulder PJI [19,25,42,43,44]. Given that some culture-negative patients had their primary surgery performed at an outside institution, it was not possible to determine the usage of antibiotics before or during the first stage, which could explain the high percentage of culture-negative cases in our cohort. Overall, the existing literature demonstrates that around 15% of shoulder PJIs are culture-negative [43,45].

This study has several limitations. The conclusions above may be limited by the retrospective nature of the present study and the high rate of exclusions due to the patients’ old age, their comorbidities, and the study’s long-term follow-up. However, regardless of the actual reinfection rate within the cohort, shoulder PJI and its treatment are associated with a high risk of mortality, especially among older patients with comorbidities [14]. Furthermore, the ROM, the VAS for pain, the SSV, the CS, and the SF-12 were not assessed preoperatively, which reduces the ability to comment on the procedure-related improvements in the functional scores. Although good outcomes were illustrated for this cohort, we could not identify the prognostic factors that contributed to the success or failure of two-stage revision of shoulder PJI due to the small sample size in an inhomogeneous population. Furthermore, despite the Delphi-based ICM criteria, there is no certain consensus on the PJI definition criteria, which largely influence the clinical course of the patients as well as the outcomes and comparability of the existing studies. Lastly, the long follow-up period is a notable strength of this investigation, providing valuable insights into the long-term outcomes of two-stage revision for shoulder PJI. Future investigations, ideally randomized clinical trials, reporting the long-term outcomes after two-stage revision are needed to further improve our understanding about its effectiveness at eliminating PJI and providing the best clinical outcomes.

## 5. Conclusions

Two-stage revision arthroplasty for shoulder PJI results in satisfactory subjective and objective outcomes, with a low overall reinfection rate. However, the high rates of mortality and failure to reimplant must be carefully considered when managing expectations in this challenging cohort.

## Figures and Tables

**Table 1:** Baseline characteristics and diagnostic subgroups for PJI diagnosis based on ICM criteria.

	ICM Diagnosis				
Characteristic	Unlikely (n = 9)	Possible (n = 3)	Probable (n = 13)	Definite (n = 5)	*p*-Value
**Gender**					
Male	2 (22.2%)	2 (66.7%)	6 (46.2%)	2 (40%)	0.52
Female	7 (77.8%)	1 (33.3%)	7 (53.8%)	3 (60%)	0.52
**Age at the time of surgery**					
(mean ± SD)	69.4 ± 9.4	69.3 ± 3.1	61.2 ± 9.8	65.8 ± 9.4	0.23
**BMI**					
(mean ± SD)	27.5 ± 4.9	27.1 ± 2	25.5 ± 2.6	27.5 ± 4.9	0.82
**Prior hardware**					
HA	3 (33.3%)	0	9 (69.2%)	2 (40%)	0.11
ATSA	3 (33.3%)	0	3 (23.1%)	1 (20%)	0.69
RTSA	0	3 (100%)	1 (7.7%)	2 (40%)	<0.001 *
Spacer at presentation	3 (33.3%)	0	0	0	0.05 *
**Elixhauser Comorbidities**					
Congestive heart failure	1 (11.1%)	0	0	0	0.49
Cardiac arrythmias	2 (22.2%)	0	0	0	0.17
Valvular disease	1 (11.1%)	0	0	1 (20%)	0.41
Peripheral vascular disorders	1 (11.1%)	1 (33.3%)	4 (30.8%)	1 (20%)	0.72
Hypertension	7 (77.8%)	2 (66.7%)	6 (46.2%)	3 (60%)	0.52
Paralysis	0	0	1 (7.7%)	0	0.74
Other neurological disorders	3 (33.3%)	0	3 (23.1%)	1 (20%)	0.69
Chronic pulmonary disease	1 (11.1%)	0	2 (15.4%)	2 (40%)	0.43
Diabetes	1 (11.1%)	2 (66.7%)	5 (38.5%)	1 (20%)	0.18
Hypothyroidism	3 (33.3%)	1 (33.3%)	1 (7.7%)	2 (40%)	0.36
Renal failure	3 (33.3%)	0	2 (15.4%)	1 (20%)	0.59
Rheumatoid disease	1 (11.1%)	0	3 (23.1%)	1 (20%)	0.75
Obesity	0	0	2 (15.4%)	0	0.72
Depression	2 (22.2%)	0	0	0	0.17
**Timing of infection**					
Acute	0	0	0	1 (20%)	0.16
Sub-acute	3 (33.3%)	0	3 (23.1%)	1 (20%)	0.29
Chronic	6 (66.7%)	3 (100%)	10 (76.9%)	3 (60%)	0.16

HA, hemiarthroplasty; ATSA, anatomical total shoulder arthroplasty; RTSA, reverse total shoulder arthroplasty. * Implies statistical significance.

**Table 2:** Summary of identified bacteria strains in cultures conducted with tissue samples from the first stage (or prior diagnostic arthroscopy).

Bacteria Identified	Number of Cases	Percentage of Total Cases
*Cutibacterium acnes*	8	26.7%
*Staphylococcus epidermidis*	8	26.7%
*Staphylococcus capitis*	2	6.7%
*Staphylococcus aureus*	2	6.7%
*Staphylococcus hominis*	1	3.3%
*Enterococcus faecalis*	1	3.3%
*Enterobacter cloacae*	1	3.3%
*Pseudomonas aeruginosa*	1	3.3%
*Streptococcus parasanguinis*	1	3.3%
*Paenibacillus pabuli*	1	3.3%
*Anaerococcus prevotii*	1	3.3%
Culture-negative	9	30.0%

**Table 3:** An overview of every patient with a clinical follow-up, showing demographics, implant design of choice at revision, and follow-up results.

Sex (F/M)	Side (R/L)	Age (Years)	Prosthesis Type (Revision)	ABD (°)	FF (°)	ER (°)	IR (Points)	VAS (Points)	SSV (%)	CS (Points)	SF-12 (Points)
F	R	78	RTSA	90	90	0	6	0	80	48	72.6
M	L	75	HA	100	120	30	4	3	95	61	92.4
M	R	72	RTSA	80	130	60	4	0	75	87.5	108.7
F	L	49	ATSA	45	75	0	0	3	30	31	56.1
M	L	65	RTSA	160	160	30	8	1	85	56.5	72.5
F	R	76	RTSA	45	45	25	8	0	25	41	86.7
F	L	79	RTSA	90	100	0	2	0	30	47	93.78
F	R	63	ATSA	100	130	35	2	0	20	69	51.7
F	L	84	RTSA	60	90	30	0	1	40	42	68.7
M	R	82	RTSA	165	165	60	2	0	70	80	98.1
M	L	79	RTSA	100	135	30	2	0	95	79.5	92.2
F	R	77	RTSA	90	120	10	2	0	50	63	78.8
M	R	63	RTSA	160	160	30	2	0	85	91.7	81.3
M	R	64	RTSA	150	140	20	4	2	65	76	79.7
F	L	58	RTSA	60	60	0	2	2	10	26	56.5
F	L	88	RTSA	70	60	20	8	0	60	47	78.7
F	L	77	RTSA	45	45	0	2	6	40	28	97.6
F *	L	97	RTSA	0	0	0	0	7	10	11	77.2
F	L	78	RTSA **	0	0	0	4	0	20	23	105.6
M	R	65	ATSA ***	110	130	10	4	4	50	84.4	72.5

F, female; M, male; R, right; L, left; HA, hemiarthroplasty; ATSA, anatomical total shoulder arthroplasty; RTSA, reverse total shoulder arthroplasty; VAS, visual analogue score; SSV, subjective shoulder value; CS, Constant score; SF-12, 12-Item Short Form Survey; IR, active internal rotation (0: lateral side of the thigh, 2: buttocks, 4: lumbosacral transition, 6: third lumbar vertebral body, 8: twelfth thoracic vertebral body, 10: between shoulder blades); ER, active external rotation; FF, active forward flexion; ABD, active abduction. * Suffered a traumatic periprosthetic fracture in follow-up. ** Revision prosthesis explanted due to persistent PJI. *** Revision prosthesis was revised with another two-stage revision arthroplasty due to persistent PJI, converting it to an RTSA.

## Data Availability

The data presented in this study are available on request from the corresponding author due to data privacy restrictions.
